# Feasibility and acceptability of the MentiParent AI chatbot for training parental reflective functioning

**DOI:** 10.1038/s41598-026-47934-4

**Published:** 2026-04-16

**Authors:** Karen Yirmiya, Elad Refoua, Alexandra Truscott, Hayley Reeve, Peter Fonagy, Zohar Elyoseph

**Affiliations:** 1 Department of Psychology, Ben-Gurion University of the Negev, Be’er Sheva, Israel; 2 Research Department of Clinical, Educational and Health Psychology, University College London, London, UK; 3 Department of Psychology, Bar-Ilan University, Ramat Gan, Israel; 4 Anna Freud National Centre for Children and Families, London, UK; 5 School of Therapy, Counseling and Human Development, Faculty of Education, University of Haifa, Haifa, Israel

**Keywords:** Generative AI, Mentalization, Parental reflective functioning, Parenting interventions, Chatbot, Digital mental health, Artificial intelligence, Psychology, Human behaviour

## Abstract

This study presents a novel generative AI-based chatbot, MentiParent, designed to enhance Parental Reflective Functioning (PRF) through interactive, simulated parent-child interactions. Addressing existing limitations of traditional PRF interventions this proof-of-concept investigation assessed the feasibility and acceptability of implementing AI-driven technology within reflective parenting support. Two pilot studies were conducted. In Study 1, sixty mental health practitioners and graduate students engaged with MentiParent, simulating realistic interactions with a virtual adolescent (“Danny”). In Study 2, thirty-six parents from the general population interacted with an updated version of MentiParent featuring a new virtual child (“Evelyn”). All participants evaluated the MentiParent usability and relevance for real-world parenting contexts on a scale of 1–7, as well as provided qualitative feedback. Both practitioners and parents rated the chatbot highly for feasibility (M_practitioners_ = 5.61, SD = 1.45; M_parents_ = 6.03, SD = 1.18) and acceptability (M_practitioners_ = 5.56, SD = 1.20; M_parents_ = 5.53, SD = 1.37), with no significant differences between groups. Qualitative feedback highlighted engaging and realistic interactions, valued feedback, and identified limitations in authenticity and technical fluidity. Ethical themes of transparency, trust, and cultural sensitivity emerged as central. Findings indicate strong feasibility and acceptability of MentiParent among both professionals and parents, supporting its potential as a scalable, ethically grounded tool for enhancing reflective parenting skills.

**Supplementary Information:**

The online version contains supplementary material available at 10.1038/s41598-026-47934-4.

## Background

Parental Reflective Functioning (PRF) describes caregivers’ capacity to understand and interpret their own and their child’s mental states, a fundamental ability underpinning secure attachment and healthy child development^1–3^. Enhanced PRF enables caregivers to respond sensitively to children’s emotional needs, thereby facilitating social learning, psychological wellbeing, and children’s emerging mentalizing abilities^4,5^. Higher PRF is consistently associated with fewer emotional and behavioral problems and improved social-emotional development in children^3,6–8^. These positive associations likely stem from more attuned parent-child interactions that foster epistemic trust and promote adaptive internal models for emotion regulation and interpersonal understanding^9–11^. Conversely, deficits in PRF or related constructs such as mind-mindedness and insightfulness correlate with psychological vulnerabilities, including attachment insecurity, difficulties in social cognition, and compromised emotional self-regulation^3,8,12–15^.

Robust evidence linking PRF with favorable child outcomes has stimulated therapeutic approaches aimed at enhancing PRF. Interventions such as Mentalization-Based Treatment for parents^16^, Reflective Parenting^17^, Minding the Baby^2,18^, and Family Minds^19^ have shown efficacy across diverse populations. These interventions typically combine psychoeducation, guided reflection, and skills practice, supporting caregivers in developing awareness of mental states and adaptive responses during challenging interactions. Frequently delivered in group settings, these programs additionally offer shared learning and peer support opportunities.

Despite promising evidence, existing PRF interventions encounter substantial barriers to scalability and accessibility. Many interventions depend heavily on highly trained professionals, significant resource investment, and rigid delivery frameworks, restricting participation for parents experiencing socioeconomic hardship, cultural marginalization, or geographical isolation^3,20^. Individual stressors further exacerbate these structural limitations, as parents experiencing chronic stress, limited social support, or unresolved trauma often find traditional psychotherapy inaccessible or ineffective^8,21,22^. Moreover, insecure attachment patterns, heightened emotional reactivity, and difficulties establishing epistemic trust can directly impede the reflective capacities these interventions aim to enhance^23,24^. Consequently, those parents potentially benefiting most significantly from PRF-focused interventions are often least likely to engage or gain access. These challenges highlight the necessity of alternative, scalable, and culturally responsive formats to facilitate PRF skill development more flexibly and inclusively.

In response to these limitations, there has been increasing interest in digital interventions offering flexible and accessible parenting support. Online interventions have demonstrated efficacy in improving parental self-efficacy and reducing child behavioral issues in controlled trials^25–27^, highlighting technology’s potential to broaden intervention access. However, most existing digital tools predominantly rely on passive psychoeducation rather than interactive and experiential learning methods. Recent advancements in Generative Artificial Intelligence (GenAI), particularly Large Language Models (LLMs), represent an emerging avenue to address these gaps. With the capability to generate emotionally nuanced and conversational interactions, GenAI systems increasingly simulate complex human interpersonal dynamics^28–30^. Furthermore, LLMs’ potential for providing real-time, in-the-moment support aligns with evidence suggesting that therapeutic skills acquisition is most effective when parents frequently practice and receive immediate feedback^31^.

Recent studies highlight GenAI systems’ robust capabilities in emotion recognition and social-cognitive reasoning, core components of reflective functioning^32^. Emerging research indicates that GenAI can surpass human performance in recognizing emotional states from verbal and nonverbal cues, accurately interpreting emotions across diverse cultural contexts^33–36^. Additional findings suggest basic emotional understanding and perspective-taking capabilities within GenAI, essential skills for mental health contexts where high PRF is crucial. Comparative studies of AI-generated and human therapist responses indicate promising effectiveness in managing psychological distress^37–39^. Moreover, LLM-based interventions demonstrate significant potential to scale mental health services, extending reach to underserved populations^40,41^. Implementation-wise, GenAI offers scalable, culturally adaptable, continuously available interventions that foster playful, risk-free environments for trial-and-error learning. Such a feature is particularly valuable for mentalization training, enabling parents to explore various response strategies without adverse real-world interpersonal consequences^42^. Given PRF’s established role in promoting emotion regulation, secure attachment, and resilience, scalable interventions enhancing PRF may contribute to the prevention of psychiatric disorders in children and adolescents^7,8^.

The MentiParent project leverages these technological advancements specifically to enhance PRF. Designed as an interactive training platform rather than a therapeutic agent, MentiParent allows parents to engage in simulated interactions with a virtual child experiencing emotional distress. This approach facilitates real-time practice and immediate mentalization theory-based feedback, addressing the acknowledged gap between theoretical knowledge and its practical application during emotionally charged interactions. By providing a safe space for experiential learning and guided reflection, MentiParent aims to reinforce emotional and cognitive processes integral to social learning, thereby increasing parents’ application of reflective skills in real-world interactions with their children. While AI-based approaches such as MentiParent offer promising opportunities to enhance accessibility and parental support, they also raise important ethical and relational considerations, including potential risks related to data privacy, the absence of human attunement or empathic nuance, and the possibility that automated guidance might be misinterpreted without professional oversight^32^. These considerations were held in mind throughout the development of MentiParent and are further reflected upon in the Discussion.

This study primarily investigates the feasibility and acceptability of this AI-driven approach rather than clinical outcomes. The current study adopts feasibility and acceptability as primary outcomes, consistent with established implementation science frameworks that identify these as critical early-stage indicators of intervention viability prior to efficacy testing^43,44^. As a proof-of-concept research, it assesses the practical implementation and acceptability of an AI-based PRF training tool among mental health practitioners from diverse backgrounds and parents from the general population. Two complementary studies were conducted: Study 1 involved mental health practitioners and graduate trainees, reflecting the early developmental stage of the tool and the need to establish theoretical and clinical acceptability before extending testing to parents. Study 2 then explored parents’ perceptions and experiences with the tool in a small pilot sample, providing preliminary insights into its real-world relevance and usability. Given that MentiParent is designed as an adjunct to therapist-led parenting interventions, the involvement of clinicians, who are likely to recommend or integrate the tool within treatment contexts, was essential. Practitioners were therefore regarded not only as evaluators but also as potential end users, ensuring that the tool’s content and process align with therapeutic principles. Furthermore, establishing feasibility represents a crucial preliminary step before controlled efficacy trials, addressing essential questions regarding the viability and ethical integration of AI-mediated reflective functioning training. Consequently, this exploratory investigation aims to evaluate MentiParent’s potential as a scalable, accessible adjunct to traditional interventions, highlighting essential ethical considerations for its responsible implementation.

## Method

### Participants

#### Study 1: mental health practitioners

A total of 60 mental health practitioners and graduate students (aged 18 years and above) participated in study 1. Recruitment occurred through professional networks, targeted email invitations, and snowball sampling within mental health organizations. Participants reported their age within predefined categories: 18–25 years (*n* = 5, 8%), 26–35 years (*n* = 19, 32%), 36–45 years (*n* = 11, 18%), 46–55 years (*n* = 16, 27%), and > 55 years (*n* = 9, 15%). Due to smaller subgroup sizes, participants were subsequently grouped broadly into two categories: ≤35 years and ≥ 36 years (see Table 1). Gender distribution included 41 female (69.5%) and 18 male (30.5%) participants. Regarding language preference, 45 participants (75%) communicated with MentiParent in English, while 15 participants (25%) chose alternative languages. Participants represented various professional backgrounds: clinical psychologists (*n* = 10, 17%), psychologists (*n* = 10, 17%), psychotherapists (*n* = 6, 10%), psychiatrists (*n* = 5, 9%), psychoanalysts (*n* = 5, 8%), and social workers (*n* = 4, 7%). Additionally, 7 participants (12%) identified as students, and 13 participants (22%) reported other mental health-related roles.

### Study 2: parents from the general population

Study 2 included 36 parents of children aged 6–21 years, recruited via snowball sampling and community social media platforms. Complete demographic data were available for 34 participants. The sample was predominantly female (*n* = 29, 85.3%), with 5 male participants (14.7%). Participants’ ages were distributed across three categories: 26–35 years (*n* = 11, 32.4%), 36–45 years (*n* = 13, 38.2%), and 46–55 years (*n* = 10, 29.4%). Most participants identified as White British (*n* = 27, 79.4%). Additional self-identified ethnicities included White Other (*n* = 3, 8.8%), Mixed/Multiple ethnic groups (*n* = 2, 5.9%), Black/African/Caribbean/Black British (*n* = 1, 2.9%), and Asian/Asian British (*n* = 1, 2.9%). Parents reported an average of 1.88 children (SD = 0.69, Median = 2.0; range = 1–3). The majority (*n* = 24, 70.6%) did not have an adolescent child, while 10 participants (29.4%) reported having at least one adolescent. Ethical approval was secured from the UCL Research Ethics Committee (Approval ID: CEHP/2024/603). All methods were carried out in accordance with relevant institutional guidelines and regulations. Informed consent was obtained from all participants prior to their involvement, and data collection remained anonymous and voluntary.

### Procedure

All participants accessed the study via a secure online link or QR code. After reviewing the participant information sheet and providing informed consent, participants completed a brief demographic questionnaire capturing age, gender, and professional role. For study 1, the chatbot was deployed using the PMFM platform, enabling user-friendly access to the Claude Sonnet 3.5 V2 language model configured with specialized prompt architecture accessible via a direct link. The chatbot’s design incorporated detailed psychological profiles for both the virtual therapist (Dr. Menton) and the adolescent character (Danny), with multilayered emotional and behavioral trajectories embedded throughout the simulation. The prompt framework defined participant roles explicitly, established precise timing for therapeutic interventions, and set comprehensive guidelines for delivering mentalization-based feedback, providing a consistent yet responsive user experience suitable for varied technological proficiencies (see Appendix 1 for a full example of a conversation with the Danny chatbot).

This simulation placed participants in the role of a parent reconnecting with a 14-year-old adolescent, Danny, following a prolonged estrangement due to family conflict. Participants engaged with Danny through a sequence of scripted conversational exchanges. Each response from Danny consisted of verbal statements paired with descriptions of non-verbal cues (e.g., body language, vocal tone). For example, Danny might say, “I don’t care what you think”, accompanied by “[crosses arms and avoids eye contact]”, thus capturing emotional complexities that challenged participants’ reflective capacities.

**Fig. 1:**
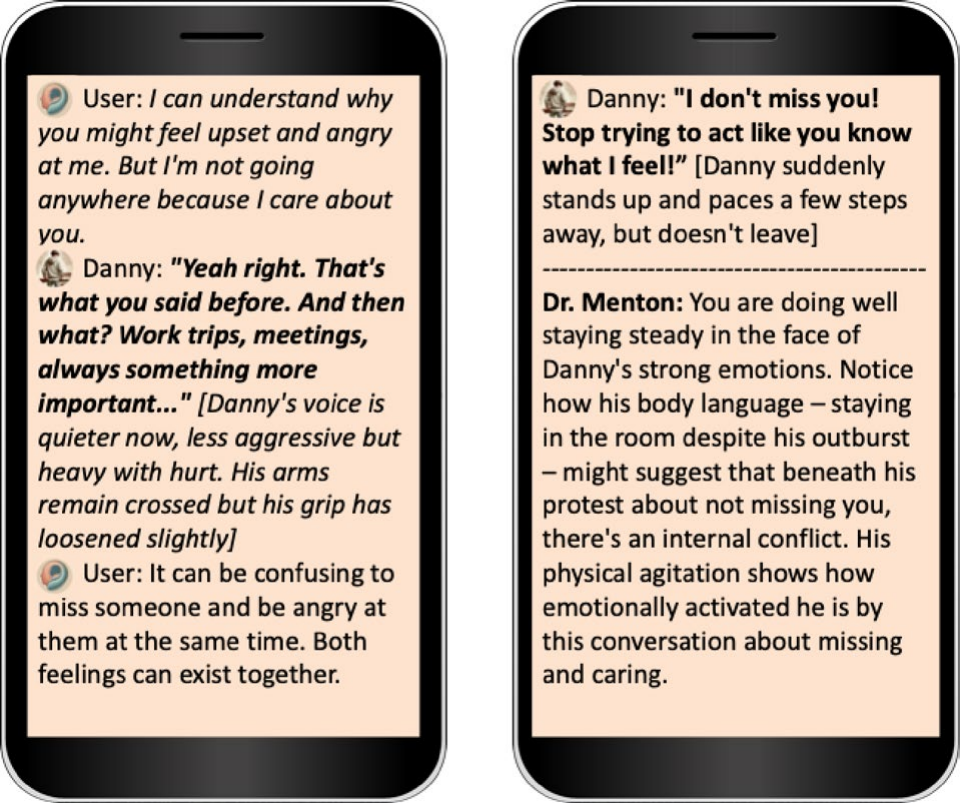
Example interaction from the “Danny” MentiParent simulation. *Note* The user, role-playing as a parent, engages in a conversation with “Danny”, a virtual adolescent exhibiting emotional distress. Throughout the dialogue, Dr. Menton, the virtual therapist, interjects periodically to provide real-time, mentalization-based feedback on the user’s responses. This structure allows users to practice Parental Reflective Functioning in emotionally charged scenarios while receiving supportive guidance.

In parallel with the simulated parent-child interactions, a virtual therapist, Dr. Menton, provided periodic interjections, delivering reflective prompts, validating emotional states, and modelling mentalization-informed strategies for conflict resolution. For instance, Dr. Menton might remark, “Danny’s tone suggests he’s feeling hurt, even though he appears angry. How do you think he’s experiencing this situation?”. Participants completed 20 structured interactions, with Danny’s responses dynamically evolving based on participant engagement. While core narrative anchors were consistent across users to ensure comparability, the chatbot generated adaptive and contextually varied responses based on participants’ input, allowing multiple conversational trajectories within the same scenario. The simulation prioritized authentic relational dynamics, mirroring the incremental and nuanced process of real-world relationship restoration rather than dramatic shifts (see Fig. 1 for an example of the interaction, and Appendix 1 for a full interaction).

Following the simulation, participants received an AI-generated reflective feedback summary from MentiParent. This feedback highlighted critical interaction moments, identifying effective acknowledgements of Danny’s emotions or areas where deeper reflective engagement was possible but missed. The feedback reinforced core mentalization principles: adopting curiosity (a ‘not-knowing’ stance), emotional validation, reflective self-awareness, perspective-taking, and affect regulation, consistent with contemporary formulations of MBT adherence and therapeutic technique^45,46^. For instance, participants received targeted suggestions for enhancing their responses to Danny’s expressions of vulnerability. Subsequently, participants completed an anonymous online evaluation hosted on Microsoft Forms, encompassing both quantitative and qualitative measures assessing MentiParent chatbot feasibility and acceptability.

To validate the initial findings from the practitioner sample (the “Danny” bot) and assess the MentiParent tool’s reception among its target end-users, we conducted in Study 2 an exploratory comparison with a new, independent sample of parents (the “Evelyn” bot). While this version maintained the core interventional structure of a challenging conversation guided by reflective prompts from the virtual therapist “Dr Menton” followed by structured feedback, it introduced a new, character named “Evelyn”. This character was designed to simulate different developmental challenges, specifically peer pressure and risky behaviors of vaping at school. A full example of a conversation with the Evelyn chatbot is provided in Appendix 2. This prototype was deployed on the secure Cesura platform, representing a technical update from the PMFM platform used in the initial practitioner pilot.

### Design and development of MentiParent

The interaction scenarios were developed drawing on the clinical experience and expertise of the authors in parenting and mentalization-based practice, aiming to reflect common parent-child dynamics, embedding emotional tension, miscommunications, and opportunities for empathy and reflective understanding. Although external stakeholders were not yet included in a formal co-design process, the development was guided by principles of practitioner-informed co-design^47^, emphasizing iterative refinement, professional consensus, and the translation of therapeutic principles into accessible chatbot dialogue. The current prototype centers on two simulated conversations, while future iterations will expand the platform to include a more diverse set of AI-based child bots representing different developmental stages, temperaments, and behavioral challenges.

### Outcomes

Perceptions of feasibility and acceptability of MentiParent were assessed in both studies with two single-item questions rated on a 7-point Likert scale (1 = not at all to 7 = very much): “To what extent did you feel that MentiParent could be part of a psychological intervention for parents?” (feasibility) and “To what extent did you feel that training with MentiParent has the potential to improve parenting skills?” (acceptability). Participants also provided open-ended feedback by responding to the following questions: “How was your experience interacting with the bot?”, “What worked well and was helpful?”, and “What worked less well and needs improvement?”. Demographic information was collected to contextualize responses and support exploratory group comparisons. For Study 1, this included participants’ age, gender, language preference, and professional background. For Study 2, parent-specific information was gathered, including age, ethnicity, gender, number and ages of children. The full Study 1 questionnaire is provided in Appendix 3.

### Quantitative analysis

For each study, we computed descriptive statistics (n, mean, SD, range) and 95% confidence intervals for feasibility and acceptability. In Study 1 (practitioners/trainees), we conducted independent-samples t-tests to compare ratings across demographic groups: age (≤ 35 vs. ≥36 years), gender (female vs. male), and language preference (English vs. other). We applied Bonferroni correction across comparisons within each demographic variable to control for multiple testing. For between-study comparisons (Study 1 vs. Study 2), we used Welch’s t-tests on the two conceptually matched items to accommodate unequal variances and sample sizes. For all t-tests, we report the test statistic, degrees of freedom, and two-tailed p-value. The significance threshold was α = 0.05 (two-tailed) and all analyses were conducted using RStudio (Version 2025.05.0 + 496).

### Content analysis

Qualitative data underwent inductive qualitative content analysis, categorizing the data in order to provide an overarching description of the feedback^48^. First, open coding assigned notes to each section of data, which were taken as each response to each question. Multiple notes were assigned where multiple ideas were contained in a response, with the aim to describe all ideas present in the data. Once open coding was complete, these notes were grouped based on conceptual similarity into categories which were able to give an overall description of the qualitative feedback. The coding was completed by the third author in discussion with the first author. Any disagreements were discussed and resolved within the research team.

## Results

### Study 1: mental health practitioners’ cohort

**Fig. 2:**
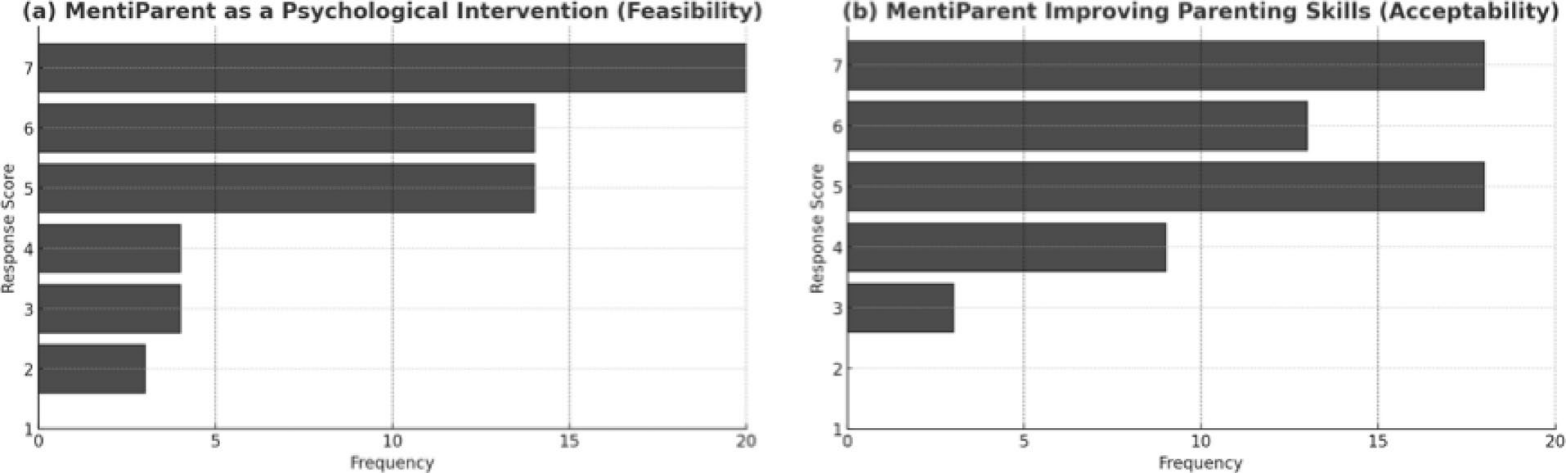
Distribution of responses on a scale of 1–7 for the potential of MentiParent to (**a**) be part of a psychological intervention for parents (feasibility) and (**b**) improve parenting skills (acceptability).

Quantitative analysis of ratings indicated strong positive perceptions regarding MentiParent’s potential (Fig. 2). For feasibility (“To what extent did you feel that the MentiParent chatbot could be part of a psychological intervention for parents?”), the mean rating was 5.61 (SD = 1.45; range = 2–7) on a 7-point scale. Similarly, for acceptability (“To what extent did you feel that training with the MentiParent chatbot has the potential to improve parenting skills?”), participants reported a mean rating of 5.56 (SD = 1.20; range = 3–7). Overall, these findings suggest favorable perceptions towards the feasibility and acceptability of the MentiParent chatbot, despite some variability.

Group comparisons indicated consistently high perceptions of MentiParent’s feasibility and acceptability across all demographic groups (Table 1). There were no statistically significant differences based on age or language preference. Although an initial gender difference emerged, with male participants providing slightly higher feasibility ratings, this difference was no longer significant following Bonferroni correction. Effect size analyses (Cohen’s *d*) indicated small effects for age and language, and a medium effect of gender on feasibility ratings, with a smaller effect observed for acceptability (see Table 1).

**Table 1:** Comparison of group differences in perceived acceptability and feasibility of MentiParent.

	Variables	Group	Mean (SD)	N	t_(df)_, *p*	Cohen’s d
MentiParent could be part of a psychological intervention for parents (feasibility)	Age	≤ 35 years	5.75 (1.33)	24	t_(58)_ = – 0.67, *p* = 0.51	0.17
		≥ 36 years	5.50 (1.56)	36		
	Gender	Male	6.11 (1.23)	18	t_(59)_ = – 2.06, *p* = 0.046	0.54
		Female	5.34 (1.51)	41		
	Language	Other	5.40 (1.24)	15	t_(58)_ = 0.68, *p* = 0.50	– 0.18
		English	5.67 (1.54)	45		
MentiParent has the potential to improve parenting skills (acceptability)	Age	Below 35	5.71 (1.27)	24	t_(58)_ = – 0.73, *p* = 0.47	0.19
		Above 36	5.47 (1.18)	36		
	Gender	Male	5.83 (1.20)	18	t_(59)_ = – 1.23, *p* = 0.23	0.35
		Female	5.41 (1.20)	41		
	Language	Other	5.40 (1.12)	15	t_(58)_ = 0.65, *p* = 0.52	– 0.18
		English	5.62 (1.25)	45		

### Qualitative results

Content analysis of mental health practitioners’ open-ended responses identified four primary categories: (1) perceived authenticity of interaction, (2) experience of Dr. Menton’s feedback, (3) views on the learning experience, and (4) user experience of MentiParent. Sub-categories captured both strengths and limitations reported by participants within these broad areas. Key insights within each category are summarized in Table 2.

**Table 2:** Participant feedback categories.

Category	Description	No. positive mentions	No. negative mentions
1. Perceived authenticity of the interaction	Feedback about how realistic the interaction felt, from the description of the situation to the responses from Danny and the participant’s own involvement	22	32
2. Experience of Dr. Menton’s feedback	Feedback about the guidance from Dr. Menton, both during the interaction and at the end of the conversation	41	5
3. Views of the learning experience	Feedback about the overall learning experience, including how engaged and challenged participants felt	26	7
4. User experience of MentiParent	Feedback about the technical experience of using MentiParent	3	20

### Perceived authenticity of the interaction

Many participants (n = 22) described the simulation as engaging and realistic, particularly noting Danny’s described body language and behavioral cues as enhancing authenticity: “*The body language piece was helpful*” and “*Danny*’*s responses were realistic*,* nothing came easily*”. Participants reflected on often unexpected emotional resonance during the interaction: *“Very surprising! I have no children but I cried several times during the exercise. It seemed like a realistic interaction and this seems like amazing practice for a situation which could arise that is highly sensitive and very difficult to simulate”.* This emotional resonance that emerged despite the simulated nature of the tool and stepping into a fictional role: *“At first I wondered how it would feel authentic when I was asked to play the role of someone else*,* but within seconds I channeled my own connection with my own child… and was all in*,* emotionally”.*

However, many participants (*n* = 32) also reported negative experiences relating to authenticity. Common critiques included interactions feeling “*repetitive*” or overly verbal, lacking important non-verbal communication aspects: “*It felt all in the verbal realm—when I would like to actively hold the space without speaking at times*”. Participants indicated needing more context on the relationship’s background: “*I was missing more background on the story so I could be more confident in my responses*”. Others described the simulation as “*intellectual rather than emotional*” and felt the pace was unrealistic compared to real-life conversations.

### Experience of Dr. Menton’s feedback

Feedback from Dr. Menton was largely perceived positively (n = 41). Participants described this feedback as “*precise and encouraging*”, “*helpful*”, and “*reassuring*”. Many valued the clarity provided regarding Danny’s emotions and appreciated guidance that “*directed me*” toward more effective responses. As one participant reflected, they *“liked getting Dr. Menton*’*s suggestions—it softened me towards Danny and his internal experience despite external appearance”.* However, a small minority (*n* = 5) found the feedback excessively critical, suggesting it be made “*more personal and get to know the participant more*”.

### Views on the learning experience

Participants frequently (n = 26) reported finding the simulation a valuable learning tool, characterizing it as “*challenging*”, “*fun*”, “*interesting*”, “*insightful*”, and “*thought-provoking*”. One participant reflected: *“It was great! Even for someone trained in therapeutic communication*,* it was challenging at times… I had to focus on my self-awareness”.* Others described how the experience prompted deep reflection and empathic resonance: *“I tried to connect with his pain and let him feel my pain. That we both hurt. Thanks for the experience. It was very meaningful to me”.* Such responses underscore the tool’s potential not only as a training resource, but also as a means of emotional self-discovery. Others appreciated the safe environment to practice difficult interactions, noting the experience encouraged adaptive thinking and perspective-shifting. Nevertheless, some concerns (*n* = 7) arose regarding the complexity and duration of the simulation. Participants suggested the required mentalization might be “*pitched quite high*” for average parents, recommended introducing “*different levels of difficulty*” and requested additional “*scaffolding and step-by-step guidance*”. Concerns about time commitment and potential emotional triggers for parents were also noted as barriers to wider engagement.

### User experience of MentiParent

While some participants (*n* = 3) found the interface user-friendly, more frequently (*n* = 16), technical issues were cited as problematic. Reported problems included difficulties in text direction (“*it’s hard to read and write from right to left*”), responses failing to register, timing inconsistencies with feedback, and confusion about participant role assignments (e.g., “*I was confused because I thought I was the mom but turned out I was the dad*”). Participants recommended improvements such as clearer guidance regarding the simulation length and providing options “*to leave the conversation at each stage*”.

Overall, these findings suggest MentiParent holds significant potential as a PRF-enhancement tool, though further refinements to authenticity, feedback personalization, user accessibility, and technical reliability are essential to maximize effectiveness and user engagement.

### Study 2: parents from the general population

Parents (*N* = 36) provided high ratings for the tool’s feasibility (M = 6.03, SD = 1.18), indication the potential of the MentiParent to be part pf a psychological intervention and a positive user experience. Parents’ mean rating for acceptability, defined as the tool potential to improve parenting skills, was M = 5.53 (SD = 1.37). Two independent-samples Welch’s *t*-tests were performed to compare conceptually similar items between the mental health practitioners (Study 1) and parent (Study 2) samples. For both feasibility and acceptability, the analyses revealed no statistically significant difference between practitioners’ and parents; ratings (*t*_(66.96)_ = 0.14, *p* = 0.889 for feasibility and *t*_(69.31)_ = 1.56, *p* = 0.123 for acceptability).

## Discussion

The present study introduces a novel application of GenAI through the MentiParent chatbot, specifically designed to enhance PRF by fostering mentalization skills. Addressing longstanding scalability and accessibility challenges in PRF interventions, this proof-of-concept investigation provides preliminary support for the feasibility, acceptability, and potential clinical utility of integrating GenAI into reflective parenting interventions. Although exploratory, the findings reveal both promising opportunities and significant challenges associated with deploying AI-driven simulations within the sensitive domain of parenting support.

Participants’ ratings of MentiParent’s feasibility and acceptability in Study 1 were consistently high across professional groups, with minimal demographic variability. Similarly, parents in Study 2 reported comparably high levels of feasibility and acceptability. The two-stage evaluation process, first with practitioners and then with parents, enabled examination of the tool’s implementation potential from both the professionals likely to recommend it and the caregivers who are its intended end-users. As MentiParent is designed as an adjunct to therapist-led parenting interventions, practitioner involvement at the initial stage was essential to ensure clinical integrity and real-world applicability. The subsequent parent evaluation yielded comparably positive ratings, with no significant differences between groups. While these findings do not establish equivalence across populations exposed to different versions of the intervention, they suggest that favorable perceptions of the tool were maintained across distinct user groups and use contexts, indicating a degree of robustness in acceptability and feasibility assessments. Involving clinical and therapeutic populations in the development and evaluation of AI-based tools is crucial for maintaining therapeutic coherence and ensuring psychological and ethical validity^32^.

Ratings exceeded thresholds typically indicative of readiness for technology adoption in clinical implementation research e.g.^49,50^. The results from Study 1 suggest a noteworthy shift in professional attitudes, historically characterized by caution towards technological innovations in mental health interventions^51^. Variability within ratings, reflected in the observed standard deviations, aligns with previous research exploring GenAI integration into clinical contexts^34,40^. Further analysis indicated that age and language preferences did not significantly influence feasibility or acceptability perceptions, highlighting potential applicability across diverse practitioner demographics. Although feasibility ratings were somewhat higher among male practitioners, this difference was not significant following Bonferroni correction; however, the medium effect size suggests a possible gender-related pattern, consistent with prior literature indicating higher levels of AI trust among men^52^.

Qualitative insights from study 1 enriched the understanding of user experiences, particularly concerning perceived authenticity and emotional resonance within the simulation. Although many mental health practitioners described the interaction as engaging, authentic, and thought-provoking, significant concerns emerged around limitations inherent to text-based interfaces, specifically the absence of embodied non-verbal communication such as facial expressions and vocal tone. These limitations resonate with prior findings emphasizing the importance of multimodal emotional information for authentic relational experiences e.g.^30,42^. Despite incorporating behavioral cues, MentiParent’s predominantly textual format constrained participants’ immersive experiences, leading some to perceive the simulation as overly cognitive rather than affectively engaging. However, these differences from an immersive, realistic experience can be a valuable part of the learning experience, as they offered additional time to consider responses and practice reflective communication without the emotional and time pressures of a real-time interaction. Such critiques highlight broader challenges in AI-mediated interventions, where fully authentic interpersonal dynamics remain difficult to replicate through current text-based technologies^36,53^.

Nevertheless, the supervisory feedback provided by the virtual therapist, Dr. Menton, was widely praised. Participants consistently characterized this feedback as precise, supportive, and beneficial for enhancing understanding of the adolescent’s emotional states. This finding underscores that even within a simulated and partially abstracted context, structured, real-time feedback based on mentalization principles was perceived by participants as supporting reflective practice and as having the potential to facilitate skill acquisition. The dynamic interplay between experiential learning and immediate, targeted guidance within MentiParent represents an advance over traditional online interventions, which typically emphasize passive psychoeducational approaches^25,26^.

Finally, mental health practitioners generally evaluated the broader learning experience positively, frequently describing it as stimulating, insightful, enjoyable, and enlightening. A notable benefit highlighted was the psychologically safe and judgement-free space provided by the AI simulation, facilitating practice of complex interpersonal skills, conditions often challenging to replicate through traditional training methods. This psychological safety, stemming from the non-evaluative AI interface, represents a key strength of MentiParent, consistent with emerging evidence on the advantages of AI-driven simulations for enhancing reflective capacity, building confidence, and promoting skill generalization.

### Potential applications and future directions for AI-based PRF training

While evidence-based PRF interventions such as Minding the Baby and Reflective Parenting demonstrate clear efficacy^9,18,54^, their reliance on in-person delivery, trained facilitators, and consistent attendance limits scalability, particularly in low-resource or marginalized settings. MentiParent builds on this foundation by offering interactive, context-responsive, and always-available PRF training, thereby addressing logistical and psychological barriers that traditional interventions cannot easily overcome. In addition to addressing access barriers, MentiParent provides innovative avenues for skill acquisition and practice. Conventional methods often rely on didactic teaching, group discussions, and limited role-play scenarios, approaches constrained by infrequent practice opportunities, inconsistent role-play quality, and delayed, generalized feedback^31,55^. MentiParent addresses these constraints by delivering a responsive, interactive environment that facilitates repeated engagement in theoretically informed scenarios accompanied by immediate, structured feedback. Such real-time formative assessment is recognized as essential for effective skill acquisition across various domains^25,26^ and appears particularly suited to developing complex mentalization skills. By harnessing GenAI capabilities to accurately interpret and respond to emotional cues^33,56^. MentiParent approximates realistic parent-child interactions. Moreover, the consistency provided by an AI-driven chatbot ensures a standardized learning environment, potentially offering advantages over the inherent variability of human role-play partners, especially beneficial for novices acquiring basic mentalization skills^35^. Consequently, MentiParent represents both a technological advancement and a methodological progression beyond traditional PRF training approaches.

Despite the promising advances AI-based interventions offer for psychological skill development, significant ethical considerations emerge, especially within sensitive relational areas such as parenting. Participant feedback highlighted broad concerns regarding trust, potential over-reliance on AI, and the necessity of human oversight. These concerns align with existing literature underscoring ethical risks associated with data privacy, confidentiality, and algorithmic bias in AI-driven mental health contexts^57–60^. Furthermore, integrating AI into parenting interventions raises fundamental questions about technology-mediated human capacities. Mentalization, inherently interpersonal, depends upon authentic emotional connection. To function effectively as a supportive tool rather than an obstacle, AI interventions must priorities the enhancement rather than replacement of genuine interpersonal engagement^61–64^. This issue is particularly salient in culturally diverse parenting contexts, where emotional expressions and interpersonal expectations may significantly differ^65,66^.

Cultural mismatches between PRF measures developed in WEIRD contexts and caregiving practices that are meaningful within specific cultural or community settings underscore the need for culturally flexible AI systems^67^. For example, future MentiParent scenarios could be adapted to reflect collectivist parenting norms or include multilingual elements that better capture culturally specific expressions of care, such as indirect communication or shared caregiving roles across extended family networks. Such flexibility is not only feasible with generative AI but essential to avoid reproducing the kinds of cultural bias and epistemic exclusion found in many conventional parenting interventions^68^. To support this, future iterations of MentiParent aim to be co-developed with local caregivers, clinicians, and community stakeholders to ensure cultural resonance and contextual fit.

An ethical framework for AI-enhanced mentalization training should emphasize transparency, clear delineation of roles, continuous evaluation, and inclusive collaboration among developers, clinicians, ethicists, and the communities served^53,69,70^. The development and deployment of MentiParent provide an early example of integrating these ethical principles in practice, although ongoing refinement will remain critical as the field matures. Building on recent calls for responsible AI in mental health^69^, MentiParent embeds principles of transparency, non-directiveness, and consent into its design. Future iterations will consider the implementation of human-in-the-loop models, wherein AI simulations are supervised by a clinician or embedded in a broader reflective parenting curriculum. Analogous to ethical deployment frameworks in digital suicide prevention chatbots^71^, these strategies may ensure that AI interventions retain therapeutic safety and user autonomy.

Lastly, the pilot findings highlight several potential applications of MentiParent in overcoming current limitations associated with traditional PRF interventions. The digital format of MentiParent could significantly expand access to mentalization training beyond geographic and logistical constraints typical of conventional programs^3^. It could also address challenges of maintaining fidelity to evidence-based methods while adapting to diverse linguistic and cultural contexts^20^. Opportunities for repeated practice with immediate feedback in a psychologically safe, low-pressure environment may reduce the anxiety associated with live supervision, thereby enhancing skill acquisition and self-confidence. Additionally, the reduced resource demands per interaction could economically facilitate multiple practice sessions, allowing users to build competencies across a broader spectrum of developmental and relational challenges.

### Limitations

Several limitations and ethical considerations must be critically acknowledged. Participants reported technical issues, repetitive interactions, and concerns regarding ecological validity due to reliance on text-based communication. Although the simulation effectively approximated aspects of PRF practice, it did not fully replicate the embodied complexity inherent in real parent-child interactions. Future technological enhancements, such as naturalistic voice synthesis or emotionally responsive avatars, may address these shortcomings, although such improvements introduce additional ethical and technical complexities^30,62^. Additionally, the current studies’ brief, one-time engagement with the MentiParent platform limits conclusions regarding sustained skill development. Mentalization skills typically necessitate repeated practice and continuous reflection over time^4^, underscoring the importance of more extensive studies featuring diverse scenarios and longitudinal designs to examine learning trajectories, retention, and real-world skill application. Furthermore, feasibility and acceptability were each assessed using single-item measures. Although pragmatically appropriate for an exploratory pilot study, this approach limits measurement reliability and the comprehensive assessment of these multidimensional constructs. Future research should employ validated multi-item instruments to evaluate these outcomes more rigorously.

A further limitation concerns the composition and comparability of the two study samples. The practitioner and parent groups represented distinct populations, with unequal sample sizes and interacted with slightly different versions of the chatbot. While this two-stage design enabled evaluation from both professional and end-user perspectives, differences in sample characteristics and chatbot prototypes may limit the direct comparability of their findings. Future research should aim to standardize platform versions across groups and include larger, demographically balanced samples to strengthen external validity. Moreover, as the field of generative AI in mental health evolves rapidly, achieving full standardization across iterations of the technology poses an inherent challenge. Rapid advancements in underlying models, interface design, and response dynamics can lead to subtle variations in user experience even within short time frames. Future research should therefore priorities transparent documentation of version updates, while recognizing that some degree of technological evolution is inevitable in this emerging area. MentiParent currently offers two scenarios (the “Danny” and “Evelyn” chatbots), which limits generalizability. Future versions will provide a broader, age-stratified and concern-specific scenario library to improve tailoring and ecological validity, while incorporating more stakeholder involvement throughout the design and refinement process.

Lastly, the use of convenience samples predominantly comprising parents, mental health professionals and graduate students may introduce selection bias towards technological acceptance, potentially limiting the generalizability of the findings. Building on this proof-of-concept phase, the next stage of research will include pre- and post-evaluation of parental competence and reflective functioning to examine potential changes associated with interaction with the tool. Future research should incorporate rigorous experimental methodologies, including control groups comparing MentiParent to conventional approaches (e.g., traditional role-play or didactic instruction), involve larger, more diverse participant samples, and expand the range and complexity of simulation scenarios.

## Conclusion

Preliminary findings from this study provide encouraging evidence for the feasibility and acceptability of integrating GenAI into PRF enhancement. The positive reception of MentiParent suggests that AI-driven simulations offer a scalable and accessible method for cultivating complex relational skills such as mentalization. Concurrently, integrating AI into sensitive interpersonal areas highlights critical considerations related to authenticity, trust, cultural responsiveness, and ethical oversight. Future research employing rigorous, longitudinal methodologies will be essential to evaluate clinical efficacy, identify potential risks, and delineate the optimal role for AI in reflective parenting interventions. By fostering key parental capacities implicated in child mental health, AI-driven interventions like MentiParent could form part of future strategies for early prevention of psychiatric difficulties.

## Supplementary Information

Below is the link to the electronic supplementary material.

Supplementary Material 1

Supplementary Material 2

Supplementary Material 3

## Data Availability

The data sets are available from the corresponding author upon reasonable request.

## Abbreviations

AI: Artificial intelligence
GenAI: Generative artificial intelligence
LLM: Large language model
PRF: Parental Reflective Functioning
SD: Standard deviation
WEIRD: Western, educated, industrialized, rich, and democratic
RCT: Randomized controlled trial
CBT: Cognitive behavioral therapy
