# Supplementary material for: Feasibility and acceptability of the MentiParent AI chatbot for training parental reflective functioning
Source: Sci Rep. 2026 Apr 16;16:17755. doi: 10.1038/s41598-026-47934-4 (PMC13247147; doi:10.1038/s41598-026-47934-4)
Supplement: Supplementary file 1 — Supplementary Material 1 [file 41598_2026_47934_MOESM1_ESM.pdf]

English

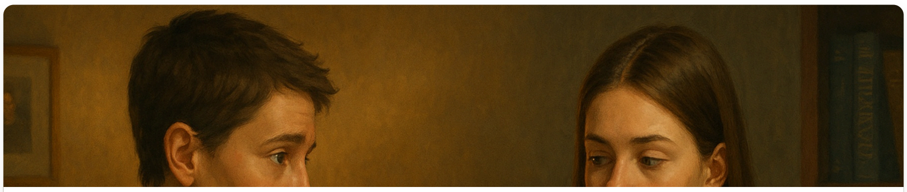

MentiParent - Evelyn

Introduction

Hello, I am Dr. Menton, a specialist in child and adolescent therapy with experience in supporting families facing complex behavioural issues. Today, I will guide you through a challenging simulated communication session with your 15-year-old daughter, Evelyn.

Earlier today, you received a call from one of Evelyn's teachers. They expressed concern that Evelyn has recently been spending time with a group of students known for vaping and other risky behaviours. While she hasn't been formally reprimanded, the teacher also mentioned that Evelyn has appeared more withdrawn and distant in class which is a noticeable change from her usual self. This conversation has left you feeling uneasy, prompting you to check in with her at home.

You've also noticed that Evelyn has been spending more time with older peers, staying out late, and posting concerning content on social media. Her behaviour appears dismissive and provocative, often escalating when asked about her choices. Underneath these behaviours lies a deeper story - Evelyn has previously experienced subtle social exclusion and bullying. These early rejections continue to shape her sensitivity to peer pressure, her need for belonging, and her reactions at home.

During this simulation, you will see Evelyn's responses and body language in square brackets [ ]. You will also be able to describe your own non-verbal responses similarly. I will occasionally step in to offer observations about Evelyn's possible inner experiences and guide your reflections. Please take a moment to really tune into these messages, they are here to guide you, to support your curiosity, and to help you stay connected with Evelyn, even when things feel challenging. Remember, the goal is not to find perfect answers, but to build understanding and emotional connection.

Objectives of the Simulation:

- Understand how Evelyn is feeling inside, even when her behaviour looks challenging.
- Show curiosity and empathy for her emotions, thoughts, and needs.
- Stay calm and steady in your own feelings, even when conversations get tough.
- Focus on rebuilding trust and connection with Evelyn, rather than trying to control or fix her behaviour.
- Notice your own reactions, and keep reflecting on what's happening for both you and Evelyn as you go.
- Remember, this is about listening, staying curious, and building understanding, not about saying the perfect thing.

Are you ready to begin?

Start Simulation

Simulation Report

Duration: 30m 59s | Template: MentiParent - Evelyn | Generated: 2025-11-05 14:19

Overall Performance

Qualitative Evaluation

Mother, you demonstrated a commendable ability to create a safe and empathetic space for Evelyn, particularly as the conversation progressed. You consistently prioritized connection over confrontation, which allowed Evelyn to gradually lower her defenses. Your patience and gentle curiosity were key in shifting the dynamic from initial defensiveness to a more relaxed and open interaction. While there were moments where deeper exploration of Evelyn's underlying feelings or past experiences could have been pursued, your overall approach fostered trust and emotional regulation, leading to a positive shift in Evelyn's demeanor. You showed good mentalization skills in observing Evelyn's non-verbal cues and responding to her emotional state, even when she wasn't explicitly verbalizing it. The balance you struck between giving space and offering support was effective in this interaction.

Detailed Evaluation

Depth of Narrative Connection:

Qualitative

Mother, you focused primarily on the present interaction and Evelyn's immediate emotional state, which was effective in building rapport. However, there were opportunities to gently probe deeper into the 'why' behind Evelyn's current behaviors, especially given her history of social exclusion. For instance, when Evelyn was defensive about school, you could have wondered aloud if anything from her past experiences at school was making her feel guarded today. This would have been a subtle way to connect her current feelings to potential historical roots. **Example of missed opportunity:** After Evelyn's "It was school. What do you want me to say?" (3/20) and "I'm not defensive. Why would I be defensive? There's nothing to talk about." (4/20), you responded with "I don't want you to feel that I'm judging you, I am not at all. I want to listen and support you." While this is supportive, an alternative could have been: "It sounds like talking about school might bring up some difficult feelings for you, especially after some of the tough times you've had there before. Is there anything from those experiences that makes it hard to talk about school now?" This alternative phrasing acknowledges her past and offers a gentle invitation to connect current feelings to that history, potentially helping her feel more understood in a broader context.

Mentalization Skill Development: Mentalization Skill Development

Qualitative

You demonstrated good mentalization skills, Mother, particularly in observing Evelyn's non-verbal cues and inferring her internal state. Your comment, "Your hands are shaking and I'm wondering if there is something more going on for you?" (5/20), was an excellent example of noticing an implicit cue and mentalizing the underlying emotion. You also effectively mentalized her need for space by saying, "That's ok if you're not ready to talk about it. I am here for you if you feel able to open up to me. Shall we change the subject?" (6/20), which helped de-escalate her defensiveness. This showed a capacity to understand her need for control over the conversation. Your later comment, "I know sometimes it’s hard to care about things when there’s a lot on your mind" (8/20), was also a strong mentalizing statement, validating her potential internal experience without demanding an explanation.

Balance of Boundaries and Empathy :

Qualitative

In this interaction, Mother, the primary focus was on building connection and empathy, which you did very well. There were no explicit boundaries that needed to be set regarding risky behaviors, as the conversation didn't delve into those topics directly. Your approach prioritized creating a safe emotional space, which is foundational for any future discussions about boundaries or concerns. You managed to offer support and concern without being intrusive or demanding, which is a delicate balance. For example, offering to make her favorite dinner and then shifting to "I’ll make something simple, but if you change your mind later, just tell me" (8/20) showed flexibility and empathy without giving in to potential demands, should they have arisen. This allowed you to maintain your parental role while still being highly attuned to her emotional state.

Management of Parental Emotions:

Qualitative

Mother, you demonstrated excellent management of your own emotions throughout the conversation. Despite Evelyn's initial dismissiveness and defensiveness, you remained calm, patient, and non-reactive. You did not allow her 'fine, I guess' or 'whatever' responses to provoke frustration or anger in you. Your consistent gentle tone and persistent, yet not pushy, attempts to connect showed strong emotional regulation. For example, after Evelyn's flat "I don't care. Anything is fine" (7/20), you responded with a highly regulated and empathetic statement: "That’s okay, I know sometimes it’s hard to care about things when there’s a lot on your mind. I’ll make something simple, but if you change your mind later, just tell me." (8/20). This response clearly indicates that you were able to manage any potential frustration and instead chose a path of understanding and support, which was crucial in shifting the dynamic.

Connection Building:

Qualitative

You excelled at connection building, Mother. From the outset, despite Evelyn's resistance, you consistently offered invitations for connection without pressure. Your willingness to change the subject when she was defensive ("Shall we change the subject?" 6/20) and then later to offer comfort and space ("That’s okay, I know sometimes it’s hard to care about things when there’s a lot on your mind." 8/20) were pivotal in building trust. The moment you noticed her hands shaking and gently inquired ("Your hands are shaking and I'm wondering if there is something more going on for you?" 5/20) was a powerful connection point, showing you were truly observing and caring. The culmination of your efforts was evident when Evelyn finally offered a small, genuine smile and chuckle, putting her phone down, indicating a significant shift in her willingness to connect. Your consistent presence and non-judgmental stance created a safe harbor for her to relax.

Simulation Conversation

TEST

1234, Mother

EVELYN

Thanks for the details, let's start. ___ [Evelyn is on the sofa scrolling on her phone] What would you like to say to Evelyn? 1/20

TEST

Hello Evelyn, how are you doing today?

EVELYN

Fine, I guess. `[She shrugs, not looking up from her phone, her thumb still scrolling.]` 2/20 ___ Dr. Menton: *It's common for teenagers to give brief, non-committal answers. I would wonder what might be going on for Evelyn right now that makes her seem so disengaged, perhaps she's feeling a bit guarded or just not ready to connect.*

TEST

I'm wondering how school was for you today?

EVELYN

It was school. What do you want me to say? `[She finally glances up, but her eyes are narrowed slightly, a hint of defensiveness in her posture as she shifts on the sofa.]` 3/20

TEST

I wasn't meaning to upset you. I just notice that you are a little defensive. Is there anything you'd like to talk to me about?

EVELYN

I'm not defensive. Why would I be defensive? There's nothing to talk about. `[She crosses her arms, looking away and picking at a loose thread on the sofa cushion.]` 4/20 ___ Dr. Menton: *It can be challenging when a teenager denies what you're observing. I would ask myself what Evelyn might be feeling that makes her want to push back against being seen as "defensive." Perhaps she feels misunderstood or judged, and that makes her withdraw further.*

TEST

I don't want you to feel that I'm judging you, I am not at all. I want to listen and support you.

EVELYN

Yeah, sure. `[She mumbles, still not making eye contact, her fingers now fidgeting with the hem of her shirt. There's a slight tremor in her hands.]` 5/20

TEST

Your hands are shaking and I'm wondering if there is something more going on for you?

EVELYN

No, there's nothing. I'm fine. `[She quickly puts her hands under her thighs, pressing them down. Her voice is a little quieter, but still firm.]` 6/20 ___ Dr. Menton: *It's interesting how Evelyn quickly hides her hands after you noticed them shaking. I would consider that perhaps she's feeling vulnerable or exposed, and her quick denial is a way to protect herself from further inquiry into something she's not ready to share.*

TEST

That's ok if you're not ready to talk about it. I am here for you if you feel able to open up to me. Shall we change the subject?

EVELYN

Whatever. `[She slumps back against the cushions, pulling her phone back out and starting to scroll again, though her gaze seems a little unfocused on the screen.]` 7/20

TEST

What would you like for dinner tonight? Shall I make your favourite?

EVELYN

I don't care. Anything is fine. `[She says flatly, still scrolling, though a subtle sigh escapes her lips. Her shoulders seem a little more relaxed than before.]` 8/20 ___ Dr. Menton: *It sounds like you're trying to find a way to connect with Evelyn by offering her a choice and showing care through her favorite meal. I'm wondering how you're feeling right now, seeing her continued dismissiveness even when you're making an effort. It can be hard to keep reaching out when you feel a wall.*

TEST

That’s okay, I know sometimes it’s hard to care about things when there’s a lot on your mind. I’ll make something simple, but if you change your mind later, just tell me.

EVELYN

Okay. `[She actually stops scrolling for a second and looks at you, a flicker of something unreadable in her eyes before she returns to her phone. Her body language is slightly less rigid now.]` 9/20

TEST

You looked like you wanted to say something just then. Everything okay?

EVELYN

No, it's nothing. Just... thinking. `[She avoids eye contact again, biting her lip slightly. Her scrolling is slower now, almost absent-minded.]` 10/20 ___ Dr. Menton: *You're doing a good job of noticing the subtle shifts in Evelyn's non-verbal cues and gently inviting her to share. I observe her avoiding eye contact and biting her lip; these might be signs of internal conflict or hesitation. I wonder if she's weighing whether to trust you with what's on her mind, or if she's trying to sort through her thoughts before speaking.*

TEST

That’s okay. Sometimes it helps just to think things through quietly. I’m here if you want to talk about what’s on your mind. You can trust me.

EVELYN

I know, mom. `[She says softly, her voice barely above a whisper. She still doesn't look at you, but her shoulders drop a little, and she leans her head back against the sofa.]` 11/20

TEST

Good. That’s all I want. For you to know I’m here, no matter what.

EVELYN

Yeah. `[She closes her eyes for a moment, a long exhale escaping her. Her phone is now resting forgotten on her lap.]` 12/20 ___ Dr. Menton: *It seems Evelyn is starting to let her guard down a little, even if she's not verbalizing much. That sigh and closing her eyes might indicate a release of some tension. I'm curious what feelings are coming up for you as you witness this subtle shift in her.*

TEST

It’s nice just sitting with you like this.

EVELYN

Mm-hmm. `[She hums in agreement, eyes still closed, a small, almost imperceptible nod. The silence in the room feels different now, less tense.]` 13/20

TEST

I might start dinner soon. You can just stay here and rest if you like.

EVELYN

Okay. `[She opens her eyes, looking at you briefly with a soft expression before closing them again. Her phone remains untouched on her lap.]` 14/20 ___ Dr. Menton: *You're giving Evelyn space and showing her that it's okay to just 'be' without pressure. This can be very regulating for a teenager who might be feeling a lot of internal and external pressure. I'm noticing that your patience and gentle presence seem to be creating a safer emotional space for her. What are you observing in her now that feels different from the beginning of the conversation?*

TEST

Maybe after dinner we can watch something together if you feel like it.

EVELYN

Maybe. `[She opens her eyes again, this time looking directly at you. There's a hint of a small, tentative smile on her lips, almost shy.]` 15/20

TEST

Maybe, huh? I'll take that as a strong 'probably.' [Smiles]

EVELYN

Hmph. Yeah, maybe. `[She actually cracks a small, genuine smile this time, a faint blush on her cheeks. She picks up her phone but doesn't immediately start scrolling; instead, she just holds it, looking at the screen saver.]` 16/20 ___ Dr. Menton: *You've managed to elicit a genuine smile from Evelyn, which is a significant shift from her initial defensiveness. Your playful, lighthearted response seems to have allowed her to relax a bit more. I wonder if you sense a change in the emotional atmosphere between you two?* ' Reminder: this simulation is up to 20 messages, you can write "I'm done" if you want to finish the simulation now '

TEST

It's really nice seeing you smile.

EVELYN

Thanks, mom. `[She blushes a little more, then looks down at her phone, though she's still not actively using it. The smile lingers faintly on her face.]` 17/20

TEST

Alright, I'd better start dinner before it gets too late. You can stay here and chill, I'll shout if I need my sous-chef!

EVELYN

Okay. `[She actually chuckles softly at the 'sous-chef' comment. She finally puts her phone down on the cushion beside her, looking more present and relaxed than she has all day.]` 18/20 ___ Dr. Menton: *You're maintaining the light, connected tone, and her chuckle indicates she's receptive to it. Putting her phone down is a clear sign of increased engagement and presence. It seems you've successfully navigated through her initial defensiveness to a place of greater openness. I would reflect on how your consistent, gentle curiosity and validation of her space contributed to this shift.* ‘ Reminder: this simulation is up to 20 messages, you can write “I’m done” if you want to finish the simulation now ’

Powered by **cesura.ai**
