# Supplementary material for: Feasibility and acceptability of the MentiParent AI chatbot for training parental reflective functioning
Source: Sci Rep. 2026 Apr 16;16:17755. doi: 10.1038/s41598-026-47934-4 (PMC13247147; doi:10.1038/s41598-026-47934-4)
Supplement: Supplementary file 2 — Supplementary Material 2 [file 41598_2026_47934_MOESM2_ESM.pdf]

Feasibility and acceptability of the MentiParent AI chatbot for training parental reflective functioning

Yirmiya K., Elyoseph, Z., Refoua, E., Truscott, A. & Fonagy, P.

Thank you for considering participaintg in the !MentiParent study

Please complete this form after you have read the Information Sheet: https://liveuclac-my.sharepoint.com/:b:/g/personal/ucjukyi_ucl_ac_uk/EauViTFBRFhDt4X0M2F0byoBb5XhiP7PVv8z4tM7ERX1ng?e=7m04bY

If you have any questions about the project, please contact Dr. Karen Yirmiya at k.yirmiya@ucl.ac.uk

1

I have read and understood the Participant Information Sheet and agree to proceed *

Yes ☐

CONSENT FORM FOR PARTICIPANTS IN RESEARCH STUDIES

Please complete this form after you have read the Information Sheet and/or .listened to an explanation about the research
Thank you for considering taking part in this research. The person organising the research must explain the project to you before you agree to take part. If you have any questions arising from the Information Sheet or explanation already given to you, please ask the researcher before you decide whether to join in. **I confirm that I understand that by ticking/initialling each box below I am consenting to this element of the study.I understand that it will be assumed that unticked/initialled boxes means that I DO NOT**

2

I confirm that I have read and understood the Information Sheet for the above study. I have had an opportunity to consider the information and what will be expected of me. I have also had the opportunity to ask questions which have been answered to my satisfaction *and would like to take part in testing the "Mentiparent" chatbot and provide feedback on its acceptability .and usefulness* *

3

I consent to participate in the study. I understand that my personal information will be used for the purposes explained to me. I understand that according to data protection legislation, .'public task' will be the lawful basis for processing *

Tick box ○

4

Use of the information for this project only *I understand that all personal information will remain confidential and that all efforts will be made to ensure I cannot be identified I understand that my data gathered in this study will be stored anonymously and securely. It will not be possible to identify me .in any publications *

Tick box ○

5

I understand that my information may be subject to review by responsible individuals from the University for monitoring and .audit purposes *

Tick box

6

I understand that by choosing to interact with the *MentiParent* chatbot, which is powered by the Claude AI language model, I agree to engage with an AI tool that operates under its own data retention policy. While my interactions will not be saved or used within this study, I acknowledge that the .AI model itself may have its own data retention practices *

7

I understand that my participation is voluntary and that I am free to withdraw at any time without giving a reason. I understand that if I decide to withdraw, any personal data I have provided up to that point will be deleted unless I agree .otherwise *

8

I understand the potential risks of participating and the support that will be available to me should I become distressed during .the course of the research *

Tick box

9

No promise or guarantee of benefits have been made to encourage you to participate *

Tick box

10

I understand that the data will not be made available to any commercial organisations but is solely the responsibility of the researcher(s) undertaking this study. *

Tick box

11

I understand that I will not benefit financially from this study or from any possible outcome it may result in in the future. *

Tick box

12

I am aware of who I should contact if I wish to lodge a complaint. *

Tick box

13

* .I voluntarily agree to take part in this study

Tick box

14

If you would like your contact details to be retained so that you can be contacted in the future by UCL researchers who would like to invite you to participate in follow up studies to this project, or in future studies of a similar nature, please let us know how (optional)

15

?What is your age

- 18 >
- 25 - 18
- 35 - 26
- 45 - 36
- 55 - 46
- 55 <
- Prefer not to say

16

Your gender

- Woman
- Man
- Non-binary
- Prefer not to say

17

What is your occupation

18

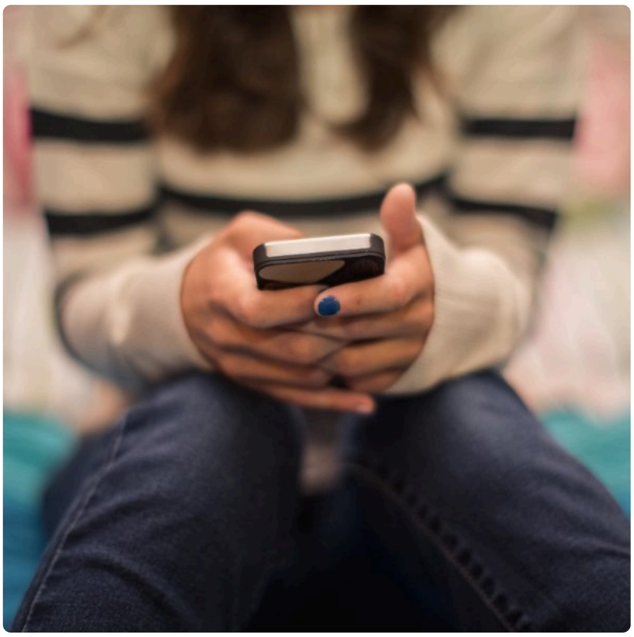

To proceed with the next step of the study, please visit the MentiParent chatbot site using the following link: https://karen2.pmfm.ai

Please do not close this window. After completing the simulation and receiving feedback from the chatbot, return to [illegible]

this page

To help us improve the bot and the feedback, we would appreciate it if you could share your responses to the following questions: On a scale of 1 to 7 how would you rate the following statements

19

To what extent did you feel that training with the *Mentiparent* has the potential to improve parenting skills

7	6	5	4	3	2	1

verv much Not at all

20

To what extent did you feel that the *Mentiparent* could be part ?of a psychological intervention for parents

7	6	5	4	3	2	1

very much Not at all

Open questions

21

?How was your experience interacting with the bot

22

?What worked well and was helpful

23

?What worked less well and needs improvement

24

Can you tell us more about your ratings? Any additional comments or feedback? Can you tell us more about your ?ratings? Any additional comments or feedback

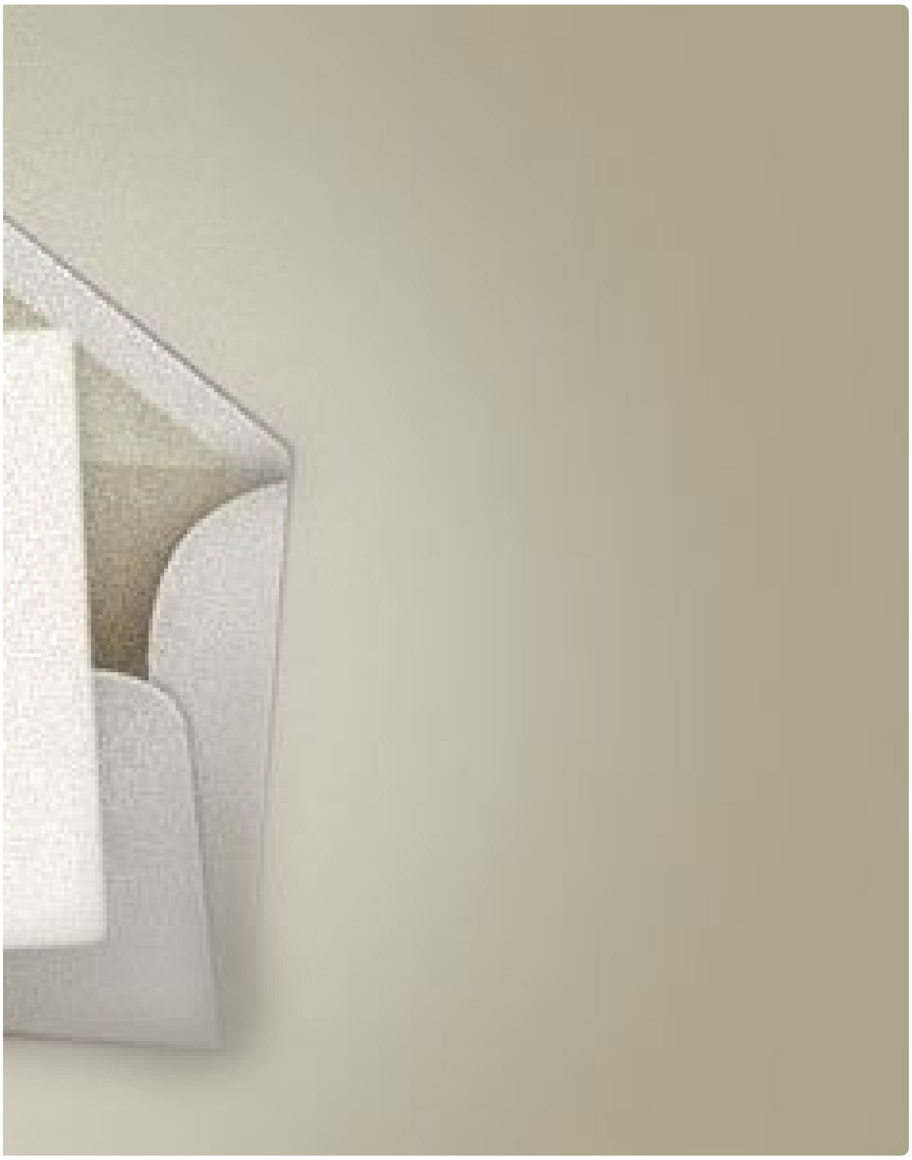

Microsoft Forms
