# Supplementary material for: Feasibility and acceptability of the MentiParent AI chatbot for training parental reflective functioning
Source: Sci Rep. 2026 Apr 16;16:17755. doi: 10.1038/s41598-026-47934-4 (PMC13247147; doi:10.1038/s41598-026-47934-4)
Supplement: Supplementary file 3 — Supplementary Material 3 [file 41598_2026_47934_MOESM3_ESM.pdf]

Share Chat

MentiParent

Feasibility and acceptability of the MentiParent AI chatbot for training parental reflective functioning

let's start in english

User
let's start in english

MentiParent
?How would you like me to address you (he/she/they)
? and which language would you prefer

User
She and English

MentiParent
.Please enter an identifier code

User
12345

MentiParent
Hello, I am Dr. Menton, a specialist in child and adolescent therapy with experience in dealing with situations of parental alienation. I will be guiding you today in a challenging, imagined communication simulation with Danny, your 14-year-old son. After the divorce, there were challenging times that included frequent work travel, tensions with the other parent, and difficulties in communication with Danny. For the past six months, contact with Danny has been almost completely cut off. Danny is now in .a state of advanced parental alienation, and you are here to visit him after a long time

During the simulation, you will see Danny's responses and his body language in square brackets [ ], and you can also describe your non-verbal responses in the same way. I will occasionally intervene with insights and explanations to help you better understand Danny's inner world and the appropriate ways .to cope with his situation
.When you feel you are finished, write 'I'm done' and we will move to the feedback stage

:The goals of the simulation are
To rebuild trust with Danny by developing a deep understanding of his emotional experience, • identifying his feelings, and providing an empathetic response to the emotions, thoughts, and needs he .expresses
To try to respond in a regulated manner to the situation despite the difficulty, to contain the • .difficulties he expresses, and not to give up on the connection

?Ready to begin

User
Yes

MentiParent
?What would you like to say to Danny

User
Hi Danny, it's lovely to see you

MentiParent
Lovely? I don't know what's so lovely about it." [He doesn't look at you, his gaze fixed on the corner" of the room. He shrugs his shoulders slightly and keeps his arms crossed tightly over his chest.]
____
1/20

User
You sound a bit unsure about being here with me. I wonder what it's like for you seeing me after such a ?long time

Feasibility and acceptability of the MentiParent AI chatbot for training parental reflective functioning

MentiParent
Why are you asking that? You weren't here for six months. It's not like you cared then." [He finally" looks at you, but his eyes are narrowed. He makes a dismissive gesture with his hand before crossing his arms again, even tighter this time.]
____
Dr. Menton: Your open-ended question was an invitation for connection, but for Danny, it might have

felt threatening. Notice how he immediately deflected by attacking your past actions. This intense anger often acts as a shield, protecting him from more vulnerable feelings like hurt or disappointment. .He is, in a way, testing whether you can stay present with his anger

2/20

User

It sounds like you felt really hurt and let down when I wasn't around. I can understand why you'd feel .angry about that

MentiParent

Don't tell me you understand. You have no idea. You're just saying that now to make yourself feel" better." [He shakes his head, looking down at the floor. He takes a small step back, increasing the physical distance between you.]

____

3/20

User

You're right, I can't fully know what it's been like for you. It sounds like it's been really painful, and .maybe hard to believe that I care

MentiParent

So what if it was painful? You were busy with your own life. You made your choice." [He looks" away, towards the window, as if something outside is more interesting. His jaw is tight, and he taps his foot on the floor impatiently.]

____

Dr. Menton: Notice the pattern here. Each time you get emotionally closer by validating his feelings (like acknowledging his pain and distrust), he pushes back. The question "So what?" is a powerful dismissal, an attempt to shut down the very feeling you just acknowledged because it's too vulnerable for him to hold. His statement "You made your choice" reflects the rigid, black-and-white thinking he's likely trapped in, where it's impossible to believe that a parent could make a difficult choice and still .love him

4/20

User

I can see why it looks that way. It must have felt like you were left on your own. I want to understand .more about what that's been like for you

MentiParent

Understand what?" [He lets out a short, bitter laugh, a scoff more than a real laugh. He finally turns" to face you, his posture challenging.] **"There's nothing to understand. You were gone. That's it. "?What's there to talk about**

____

5/20

User

It sounds like for you, it really feels final. Like once I was gone, that was the end of it. I wonder if it's .hard to even think there could be anything left to understand

MentiParent

Stop it. Just stop talking like that. It's not 'final for me,' it's just a fact. You weren't here." [He" frowns, a flicker of confusion crossing his face for a split second before it hardens again. He takes a step to the side, putting a small table between you and him, creating a physical barrier.]

____

Dr. Menton: Your attempt to wonder about his internal world was very insightful. Notice his immediate and forceful rejection: "Stop talking like that." For him, treating his experience as a 'feeling' or 'perspective' might feel like you're invalidating what he holds as an undeniable, factual truth. By insisting 'it's just a fact,' he's trying to shut down the very space for interpretation and feeling that you .are trying to open, because that space feels dangerous and confusing for him right now

6/20

User

I get it — you don't want me to put it into words right now. I'll just stay here with you. You don't have to .explain anything

MentiParent

Danny remains silent. He doesn't move away, but he doesn't move closer either. His arms are still] **"..."** crossed, but the tension in his shoulders seems to have eased just a little. He glances at you quickly, a [.fleeting look, before his eyes dart back to the floor

____

7/20

User

You don't have to say anything, but I noticed you looked over just now. I wonder what that moment was .like for you

MentiParent

I wasn't looking at anything. Just… whatever." [He looks away sharply, rubbing the back of his neck, a" gesture that seems both annoyed and a little self-conscious. He shifts his weight from one foot to the other.]

Dr. Menton: I am struck by your patience and your ability to notice such a small, subtle cue like that fleeting glance. It takes a lot of emotional strength to stay so attuned when met with such rejection. That glance was likely a crack in his defensive armor, a moment of involuntary connection or curiosity. Your gentle question, while well-intentioned, may have felt too exposing for him, prompting an .immediate denial to seal that crack and reassert his protective anger

8/20

User

.Sure, that's fine. I wasn't trying to pry — I just want you to know I'm here, even if you don't want to talk

MentiParent

I don't need you to 'be here'. I was fine without you for six months." [His voice is quieter now, less" aggressive, but still laced with bitterness. He walks over to the window and stares out, his back mostly to you.]

9/20

User

.I believe you've been managing, Danny. You've had to be strong, even if it's been hard

MentiParent

Who said it was hard? It wasn't hard." [He turns slightly, his profile visible. There's a slight tremor in" his voice when he speaks, despite the defiant words. He quickly clears his throat.]

Dr. Menton: It must be so difficult to hear him deny his struggle, especially when your words come from a place of care. Your acknowledgment of his strength was an empathetic move, but for Danny, admitting it was "hard" would mean admitting he needed you, a feeling he is fiercely protecting himself from. That tremor in his voice is a significant clue; it's a physical betrayal of the tough facade he's .trying to maintain, a sign that your words did land on a much more vulnerable part of him

.Reminder: You can write 'I'm done' if you wish to finish

10/20

User

.Alright, Danny. I won't push. You've been doing what you needed to cope, and I respect that

MentiParent

Danny doesn't say anything. He stays looking out the window, his back to you. After a long moment,] … his arms slowly uncross and he shoves his hands into his pockets, leaning his forehead against the cool [.glass of the windowpane

----

11/20

User

.You don't have to say anything, Danny. I'm just really glad to be here with you

MentiParent

Why? So you can leave again?" [The question is quiet, almost a mumble, directed more at the" window than at you. He doesn't turn around.]

Dr. Menton: I can imagine how much that quiet question might hurt. It's so different from the loud anger before. This feels like the heart of the matter, doesn't it? Underneath all the layers of defiance and anger, this is the core fear: "If I let you in, you will hurt me again." The fact that he's asking this, even in a mumble, is a huge step. He's momentarily stepping out of the defensive "I hate you" position .and into the vulnerable "I'm scared you'll abandon me" position. How you respond to this fear is crucial

12/20

User

I can hear how scared you are that I'll leave again. I know I hurt you before, and I understand why it's .hard to believe I'll stay

MentiParent

I'm not scared." [His voice is low and unconvincing. He pushes himself away from the window glass but" stays nearby, looking down at his feet. He scuffs his shoe on the floor.] **"It's just… what happened. ".People leave. That's what they do**

13/20

User

[illegible]

.It sounds like you've learned that people can't be trusted to stay. That must have hurt a lot

MentiParent

Whatever. It is what it is." [He shrugs, a gesture that's meant to look casual but seems heavy, weighed" down. He still won't meet your eyes, but he's no longer turned completely away. He's now standing at an angle to you.]

Dr. Menton: It's so painful to see a child adopt such a cynical, resigned worldview like "people leave." You're doing a remarkable job staying with the feeling underneath his words, naming the hurt of learning not to trust. His response, "It is what it is," is a classic defense mechanism for a teenager grappling with overwhelming feelings. It's an attempt to sound tough and unaffected, but the heaviness of his shrug and his averted gaze tell the real story: the hurt is there, it's just too big to acknowledge .directly right now

14/20

User

You say it's just what it is, but I can hear how heavy that sounds, Danny. It feels like it's been really hard .to live with

MentiParent

He remains silent for a noticeable stretch of time. He swipes at his eye quickly with the back of his] ... **You don't know"** [.hand, as if brushing something away, though he keeps his head turned from you **anything about it."** [The words are a reflex, a weak attempt to push you away, but his voice is thick with unshed tears, robbing them of their usual force.]

.Reminder: You can write 'I'm done' if you wish to finish

15/20

User

I don't want to pretend I know everything, but I can see this matters to you so much. You don't have to .go through it alone now

MentiParent

You say that now." [He finally turns to face you, but his eyes are fixed on a spot on the floor" somewhere between you. He wraps his arms loosely around his middle, as if holding himself together. The defiance is gone, replaced by a quiet, fragile sadness.]

Dr. Menton: I can feel the weight of that question, "You say that now." It must be incredibly painful to have your sincerity questioned when you are offering your whole heart. And yet, this is the most vulnerable he has been; he is no longer just showing you his anger, he is showing you the deep wound of broken trust. He's not just pushing you away; he's asking, without being able to say the words, if this .time will truly be different

16/20

User

.It's okay to doubt me. I get that. I'll keep showing up, even if it takes time for you to believe it

MentiParent

He doesn't respond verbally. He just stands there, his arms still loosely around his middle. After a] ... long moment, his gaze flickers up to your face for just a second before dropping back to the floor. His [.shoulders seem to lose a tiny bit of their tension

----

17/20

User

.I can see this is hard for you. I'm really glad you're here with me right now

MentiParent

I'm still here, aren't I?" [He mumbles the words, not looking up from the floor. He takes a small, almost" imperceptible step back and shoves one hand into his pocket, while the other arm stays loosely across his stomach.]

.Reminder: You can write 'I'm done' if you wish to finish

18/20

User

.You are. Thank you for staying, that really matters

MentiParent

He doesn't answer. He hesitates for a moment, then walks over to the edge of his bed and sits down,] ... [.keeping his gaze directed towards the floor. He hunches over slightly, resting his elbows on his knees

----

19/20

User

.You've shown a lot of strength today, Danny. Thank you for letting me be here with you

[illegible]

MentiParent

I'm... tired." [He says it quietly, almost to himself. He hunches over a little more, letting out a long, slow" breath. For the first time, his body looks less like it's bracing for an attack and more like it's just... exhausted.]

20/20

Type your message

Request Support
